# Gas identification with graphene plasmons

**DOI:** 10.1038/s41467-019-09008-0

**Published:** 2019-03-08

**Authors:** Hai Hu, Xiaoxia Yang, Xiangdong Guo, Kaveh Khaliji, Sudipta Romen Biswas, F. Javier García de Abajo, Tony Low, Zhipei Sun, Qing Dai

**Affiliations:** 1 Division of Nanophotonics, CAS Center for Excellence in Nanoscience, National Center for Nanoscience and Technology, 100190 Beijing, China; 2 University of Chinese Academy of Sciences, 100049 Beijing, China; 3 Department of Electrical and Computer Engineering, University of Minnesota, Minneapolis, MN 55455 USA; 4 ICFO-Institut de Ciencies Fotoniques, The Barcelona Institute of Science and Technology, 08860 Castelldefels (Barcelona), Spain; 5 ICREA-Institució Catalana de Recerca i Estudis Avançats, Passeig Lluís Companys 23, 08010 Barcelona, Spain; 6 Department of Electronics and Nanoengineering, Aalto University, Tietotie 3, FI-02150 Espoo, Finland; 7 QTF Centre of Excellence, Department of Applied Physics, Aalto University, FI-00076 Aalto, Finland

## Abstract

Identification of gas molecules plays a key role a wide range of applications extending from healthcare to security. However, the most widely used gas nano-sensors are based on electrical approaches or refractive index sensing, which typically are unable to identify molecular species. Here, we report label-free identification of gas molecules SO_2_, NO_2_, N_2_O, and NO by detecting their rotational-vibrational modes using graphene plasmon. The detected signal corresponds to a gas molecule layer adsorbed on the graphene surface with a concentration of 800 zeptomole per μm^2^, which is made possible by the strong field confinement of graphene plasmons and high physisorption of gas molecules on the graphene nanoribbons. We further demonstrate a fast response time (<1 min) of our devices, which enables real-time monitoring of gaseous chemical reactions. The demonstration and understanding of gas molecule identification using graphene plasmonic nanostructures open the door to various emerging applications, including in-breath diagnostics and monitoring of volatile organic compounds.

## Abstract (web summary)

Identification of gas molecules is crucial in healthcare and security applications. Here the authors achieve label-free identification of SO_2_, NO_2_, N_2_O, and NO gas molecules by detecting their rotational-vibrational modes using graphene nanoribbon plasmons.

## Introduction

Label-free identification of gas molecules is very desirable for applications such as high-quality chip fabrication in semiconductor technology^1^, detection of explosives^2^, and medical diagnostics^3,4^. For example, for diagnostics, the presence of NO in the breath of patients is typically associated with chronic obstructive pulmonary disease^5,6^, while isopropanol^7,8^ and ammonia^9,10^ in the breath of patients are normally linked to lung cancer and renal failure disease, respectively. Recently, the sensitivity of electrical devices has been improved to the single-molecule level using nanomaterials^11–17^. In addition, refractive-index sensing of gas molecules using plasmons are also approaching very high sensitivity^18–21^. However, the identification of trace gases has been fundamentally hindered. This is mainly due to the fact that the intrinsic detection variations (e.g., differences in electrical conductivity or resonance wavelength) in these devices are not directly correlated with the components and structures of the gas molecules^22–26^, and therefore, these methods are unable to identify molecular species without molecular labels (Supplementary Table 1 and 2).

Recently, graphene-plasmon-based surface-enhanced infrared absorption (SEIRA) spectroscopy^27–33^, relying on the coupling of molecular vibrational modes with graphene plasmon resonances, has been shown to provide a label-free method to identify trace solid-state molecules, such as protein monolayers^28^ and nano-sized polymer films^29,30^. In particular, ultrasensitive graphene plasmons have been demostrated to be able to detect 0.6 nm thickness of perylene-3,4,9,10-tetracarboxylic dianhydride (PTCDA) and a chemical bond vibration in acetone and hexane vapor^32^. However, there is a technologically important challenge to extend solid-sample sensing to gas sensing with SEIRA:^19,20,34,35^ the dielectric response of gases at ambient pressure is >4 orders of magnitude weaker than that of solid molecular layers due to the difference in density^18,19^. For example, a 500-μm-thick NO_2_ layer with a concentration of 1000 ppm has the same optical density (~0.25%) as an ~10-nm-thick poly(methyl methacrylate) layer^29,30^. Furthermore, the large spatial mismatch between the evanescent plasmon field (~tens of nanometers) and dispersed gas molecules limits the detection region to the immediate surroundings of the graphene layer, thus imposing another serious constraint to the applicability of graphene plasmons to gas sensing. However, if one could redistribute these gas molecules closer to the graphene surface (e.g., through adsorption, optical forces, or dielectrophoresis forces), it might be possible that the additional enhancement due to plasmonic light confinement can reveal the molecule vibrational modes.

In the present study, we identify gas molecules using graphene plasmons. The rotational-vibrational modes of the gas molecules NO_2_, N_2_O, NO, and SO_2_, which are generally important in environmental and military monitoring applications, as well as in medical diagnostics, are unambiguously detected and identified using the designed graphene nanostructures. This result relies on the adsorptive redistribution of the gas molecules on the graphene surface (equivalent to amplifying the gas concentration), hence facilitating the interaction between ultra-confined graphene plasmons and gas molecules. Our theoretical analysis reveals that the adsorbed gas-molecule layer (about 800 zeptomole per μm^2^ for <1 nm thickness) on the graphene structure, in conjunction with the strong field confinement associated with the plasmons, is critical for effectively detecting and identifying gas molecules. In addition, our graphene plasmonic sensors also successfully perform real-time monitoring of gas molecules during chemical reactions with a fast response time (<1 min).

## Results

### Graphene nanoribbon devices for gas identification

**Fig. 1:**
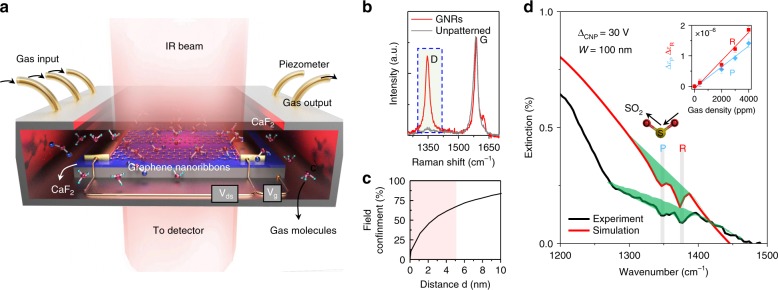
The graphene plasmon device for gas identification. **a** Experimental scheme of our device. A metal chamber with a piezometer was used for precise control of gas parameters. Plasmons in a graphene ribbon array were excited using an incident infrared beam and tuned in situ by electrostatic doping through a gate voltage (*V*_g_). The plasmon resonances were coupled with molecular excitations, thus probing the rotational–vibrational spectral fingerprints of gas molecules. **b** Raman spectrum of the graphene nanoribbons (GNRs) compared with one of an unpatterned graphene sheet. **c** Plasmonic field confinement of a typical GNR with ribbon width of 70 nm. **d** Experimental (black curve) and simulated (red curve) extinction spectra of GNRs for SO_2_ gas identification. The molecular responses on the plasmonic peaks are marked with solid green areas. The vertical gray lines indicate the rotational-vibrational modes (P, R) of SO_2_. The schematic of the vibrational mode is indicated with arrows in the central inset. The graphene ribbon width is 100 nm with a filling factor of 80%, Δ*V*_CNP_ of 30 V, and SO_2_ of 4000 ppm at 1 atm. The simulation adopts an effective ribbon width of 70 nm and Fermi energy of 0.3 eV to best fit the experimental spectra. Upper inset: oscillator strength vs concentration for the P and R modes of SO_2_. The differences between experimental and simulated spectra may originate in a narrower ribbon width and lower *E*_F_ of the fabricated nanoribbons

Figure 1a illustrates the experimental setup. A home-made IR-transparent gas chamber was designed for measuring transmittance and performing IR spectroscopy. The chamber was equipped with a high-precision piezometer and a flowmeter to precisely control the gas input. The graphene sensors were composed of connected nanoribbon arrays on a transparent IR substrate and were mounted inside the gas chamber (Supplementary Figure 1 & Methods)^30^. The graphene nanoribbon arrays were designed to have widths (*W*) in the 25–100 nm range with a high filling fraction of up to 90% to achieve strong plasmon-field enhancement over a broad mid-IR spectral range (Supplementary Figure 2). Raman characterization of the graphene nanoribbons (Fig. 1b) reveals large increases in the D peak compared to an unpatterned graphene sheet due to the vast amount of edges in the nanoribbon structure (Supplementary Figure 3).

Graphene plasmons are characterized by ultrahigh mode confinement, which can enhance the interaction between their associated evanescent field and adjacent gas molecules. In addition, this effect alleviates the need for a large volume of gas molecules for detection. Our simulation results shown in Fig. 1c suggest that the evanescent plasmon field extends ~λ_p_/4π outside of the graphene (for 1/e intensity decay), where *λ*_p_ is the plasmon wavelength in extended graphene. And for ribbons we have approximately *λ*_p_ = ~2×width (i.e., the ribbons spans ~half the plasmon wavelength), so the plasmon intensity extends a distance ~0.2×width, and ~60% of the plasmon energy is confined to a ~5-nm distance from the graphene surface (see Supplementary Note 4 for details). Therefore, in principle, a thin layer of gas molecules close to the graphene nanoribbons is sufficient for detection.

### Gas identification with high detection efficiency

Gas detection and identification measurements with our graphene nanostructures were performed by recording their IR transmission spectra using Fourier transform infrared spectroscopy (FTIR). The obtained extinction spectra *η* can be described as 1-*T*_*V*g_/*T*_CNP_, where *T*_*V*g_ and *T*_CNP_ represent the transmittance measured with an applied gate voltage *V*_g_ and at the charge neutrality point (CNP) of the graphene nanoribbons, respectively (Supplementary Note 4 and Note 2). The corresponding Fermi energies at different *V*_g_ are calculated and presented in the Supplementary Information (Supplementary Figure 5 and Note 3). We note that we employed a gas chamber that had a width of 7 mm, so that the signal coming from gas molecules, far from the graphene, was significant if we did not normalize to the neutrality point. By using this in situ electrical tuning method, the background signals, including the substrate and gas molecules, were removed. The measured extinction spectra are only the contribution which arises from the evanescent plasmonic field and its influence on molecules within a few nanometers from the graphene. This provides evidence in this work that the dominant sensing role resides in the graphene plasmons. We remark that similar results should be obtained with a thinner gas chamber, as the density of the molecule layer near graphene should only depend on molecule concentration in the gas phase, and not on the actual size of the chamber. Hence, we envision an future realistic implementation of practical devices using a sub-micron-thick gas chamber, with a total gas volume in the picolitre range.

When the chamber did not contain gases (i.e., vacuum), there was only one prominent plasmonic peak in each extinction spectrum of our plasmonic devices. The resonance peaks could be tuned within the 900–2000 cm^−1^ spectral range by varying the ribbon width or *V*_g_ (Supplementary Figure 6). When gas was pumped into the chamber, typical gas molecular signatures showed up as sharp dips in the broad plasmonic resonance peaks. A full extinction spectrum for detection of SO_2_ is presented in Supplementary Figure 7 as an example, whereas the experimental results within the frequency range of 1200–1500 cm^−1^ are shown in Fig. 1d (black curve). Clear dips are highlighted with green solid filling and appear in pairs at 1347 and 1374 cm^−1^, which can be confidently assigned to rotational-vibrational absorption features of SO_2_ molecules. A pair of molecular modes are a typical feature of gas molecules due to coupling of their rotational and vibrational modes, which results in a high-energy branch (R, 1374 cm^−1^ for SO_2_ molecules) and a low-energy branch (P, 1347 cm^−1^ for SO_2_ molecules)^36,37^.

We performed numerical simulations for a comprehensive understanding of the experimental results. A combination of the transfer-matrix method and COMSOL simulations was utilized to compute the electromagnetic response of our device (see Methods and Supplementary Note 4 for details). The graphene response is described using the Drude model^38,39^, and an optical-effective ribbon width (*W*_eff_) is introduced to account for pressumably inactive edges^40^. The dielectric permittivities of gases were retrieved from the experiments and fitted to a good approximation as the sum of Lorentzian P and R modes^37^ contributions,

$${\it{\epsilon }}_g = 1 + \mathop {\sum }\limits_i \left( {\frac{{\Delta {\it{\epsilon }}_{{\mathrm{P}},i}\left( C \right){\mathrm{\Omega }}_{{\mathrm{P}},i}^2}}{{{\mathrm{\Omega }}_{{\mathrm{P}},i}^2 - \omega ^2 - i\gamma _{{\mathrm{P}},i}\omega }} + \frac{{\Delta {\it{\epsilon }}_{{\mathrm{R}},i}\left( C \right){\mathrm{\Omega }}_{{\mathrm{R}},i}^2}}{{{\mathrm{\Omega }}_{{\mathrm{R}},i}^2 - \omega ^2 - i\gamma _{{\mathrm{R}},i}\omega }}} \right)$$

where the sum index *i* runs over the P and R pairs of spectral positions ${\mathrm{\Omega }}_{{\mathrm{P}}\left( {\mathrm{R}} \right),i}$, widths $\gamma _{{\mathrm{P}}\left({\mathrm{R}} \right),i}$, and oscillator strengths $\Delta {\it{\epsilon }}_{{\mathrm{P}}\left( {\mathrm{R}} \right),i}$. The values of these parameters were obtained by performing a series of FTIR measurements (Supplementary Figure 8) for varying gas concentrations, *C*. This yielded absorption spectra that can be well described by $A = 1 - {\mathrm{exp}}( - 2\Im \left\{ k \right\}d)$, where *d* = 7 mm is the gas chamber height, $k = (2\pi /\lambda _0)\sqrt {{\it{\epsilon }}_g}$, and *λ*_0_ is the free-space wavelength. The extracted values of $\Delta {\it{\epsilon }}_{{\mathrm{P}}\left( {\mathrm{R}} \right),2}(C)$ exhibited a linear dependence with the measured concentration (inset of Fig. 1d). We first assume that the SO_2_ gas molecules are distributed uniformly in the chamber. However, there are no noticeable dips in the calculated extinction spectra if this is the case, which indicates negligible electromagnetic interaction between the SO_2_ gas molecules and graphene plasmons due to the extremely small gas dielectric function for the tested concentrations. To achieve the dip strength observed in the experimental spectrum (black curve, Fig. 1d), the SO_2_ molecule concentration within the plasmonic near-field is expected to be much higher.

We thus attribute the experimentally observed molecular features in the spectrum to the accumulation of gas molecules on the graphene surface through physisorption, which is reasonable for graphene, especially for patterned graphene nanostructures^14,41^. We then considered an adsorbed molecular layer thickness *d*_*l*_ = 1 nm (i.e., within the effective plasmonic near-field, Fig. 1c) and an effective gas concentration *C*_*l*_. To fit our experimental data, we used a *C*_*l*_/*C* ratio of 5000. The fit results are shown in Fig. 1d (red curve) and further details are provided in Supplementary Figure 7. Note that this simulation is also supported by an analytical model (details in Supplementary Note 5). We stress that although our estimated value of the adsorbed gas concentration *C*_*l*_ was much larger than *C*, it was still a very small value, roughly corresponding to 800 zeptomole gas molecules adsorbed per every 1 μm^2^ graphene area (i.e., ~0.5 molecules per nm^2^) (see Supplementary Note 6 for details). This adsorption density is similar to what is found in water on clean surfaces, and we presume that it is related to the polar nature of the molecules under consideration (the permanent dipoles of CO, NO, NO_2_, N_2_O, and SO_2_ molecules are 0.110 D, 0.159 D, 0.316 D, 0.161 D, and 1.633 D, respectively), which produces an increase in image attraction comparable to that of water (permanent dipole 1.85 D), thus permitting nearly full monolayer coverage of the surface. Furthermore, besides the above-mentioned adsorption mechanism related to polar molecules, the large amount of edge defects and dangling bonds in the graphene nanoribbons could also help to trap gas molecules, as the adsorption energy of graphene with divacancy defects is about one order of magnitude higher than that of unpatterned graphene^41–44^. Through the mapping of the Raman D peaks, we estimated a defect density of 10^5^ μm^−2^ (see Supplementary Figure 3 and Note 1)^45^. In principle, these defects could also adsorb a gas layer of concentration up to 0.2 molecules per nm^2^ if each defect adsorbs two molecules^46^.

### Real-time gas identification

**Fig. 2:**
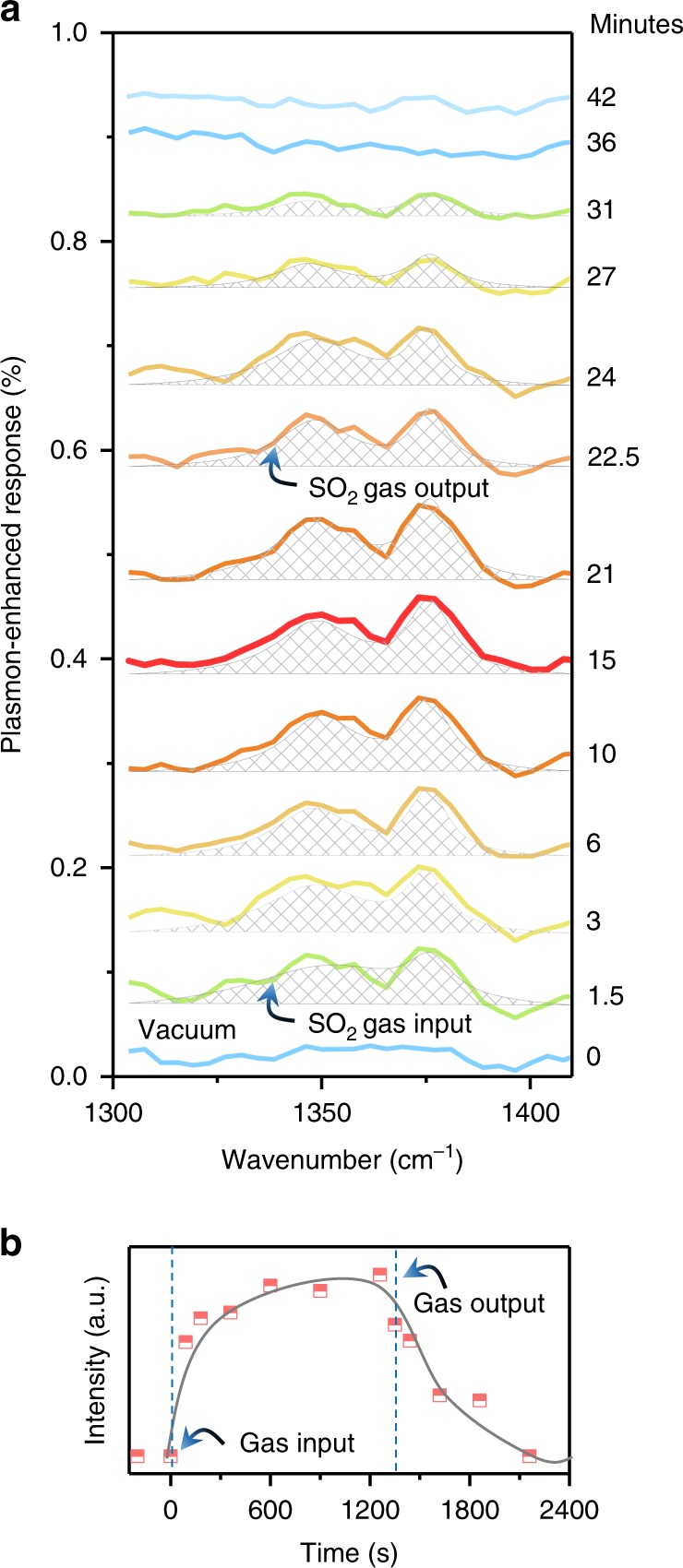
Real-time gas identification. **a** Real-time plasmon-enhanced rotational-vibrational mode response of SO_2_ during a full gas enter-exit cycle (data extracted from the extinction measurements reported in Supplementary Figure 10). Spectra were recorded at the times indicated on the right. **b** Kinetic plot of plasmon-enhanced SO_2_ signal intensity, defined as the integrated peak area in **a** as the SO_2_ gas enters and exits the chamber

We next investigated the real-time responses of our devices in detail. A series of extinction spectra were recorded while SO_2_ gas was dosed in and then washed out from the chamber. The plasmon-enhanced response dynamics shown in Fig. 2a are extracted from the original extinction spectra (Supplementary Figure 9) following the method described in Supplementary Figure 10 and Note 6. As shown, the prominent peaks of the P and R modes started to be discernable in the extinction spectra recorded 1.5 min after SO_2_ gas was introduced into the chamber. This suggests that the SO_2_ molecules entered the chamber and redistributed within 1.5 min to a detectable amount of physisorption of gas molecules on the graphene layer. Subsequently, the detected signal continued to increase and reached a maximum after 15 min. This indicates that the concentration of physisorption of gas molecules on the graphene device is peaked after 15 min. Next, pure N_2_ gas was introduced instead of SO_2_, which caused a gradual decrease in the plasmon-enhanced IR response of SO_2_, indicating desorption of SO_2_ molecules. Specifically, the signal intensity decreased by half in ~5 min, and no SO_2_ molecular signal was detected after 20 min. These two processes are clearly visualized in the dynamic plot (Fig. 2b) of the signal intensity (i.e., the peak area). The signal intensity increased sharply as the gas was pumped in, revealing fast physisorption kinetics. The rate of increase then decreased, pointing to the diffusion of gas molecules in the chamber and physisorption of the gas molecules on the graphene surface, slowing down to reach a dynamical equilibrium between physisorption and desorption. Fast desorption was also observed in Fig. 2b. Because performing a measurement for each spectrum required ~0.5 min, the real physisorption and desorption dynamics may be faster than the signal changes in the spectra. Nevertheless, the results clearly demonstrate that our devices can perform real-time monitoring of gas molecules and are reusable with N_2_ flow, which removes physically adsorbed molecules.

We also measured the extinction spectra of SO_2_ gas at different concentrations (2000, 4000, and 6000 ppm). The integrated areas of these plasmon response signals are recorded as a function of SO_2_ concentration. The devices had a near-linear response when monitoring the gas concentration, as shown in Supplementary Figure 11 and Note 8. The near-linear fit implies that the adsorption of SO_2_ molecules on the graphene surface at these gas concentrations was below the saturation threshold for graphene gas adsorption as previously discussed.

### Identification of gas molecules

**Fig. 3:**
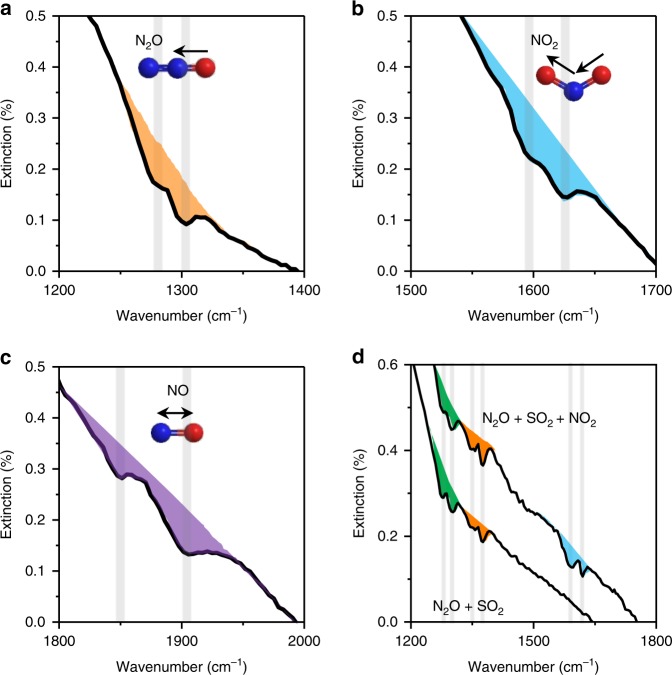
Identification of different nitrogen oxides. **a–c** Extinction spectra of graphene in the presence of N_2_O, NO_2_, and NO, respectively. The rotational–vibrational modes are marked with vertical lines. **d** Extinction spectra of graphene in the presence of two gas mixtures, one consisting of SO_2_ and N_2_O, and the other of SO_2_, N_2_O, and NO_2_. The graphene ribbon widths in **a**–**c** were 80, 60, and 40 nm, respectively, with a filling factor of 90%, Δ*V*_CNP_ of 30 V, and concentration of N_2_O of 8000 ppm, and NO_2_ and NO of 4000 ppm at 1 atm

Nitrogen oxides (i.e., NO, NO_2_, and N_2_O) were employed to show the key advantage of using graphene plasmons for identification of similar gas molecules, which still remains a challenge for sensors based on electrical methods, as the adsorption of these molecules results in similar changes in resistance (see details in Supplementary Figure 12). Figure 3a–c displays the plasmonic responses of NO, NO_2_, and N_2_O gases, respectively. The full extinction spectra are shown in Supplementary Figure 13. In each extinction spectrum, the molecular responses appear as a pair of dips, which can be assigned to the rotational-vibrational modes of the gases, as indicated with vertical gray lines. Therefore, we can clearly identify these nitrogen oxides from their rotational–vibrational fingerprint peaks. Moreover, these gases can also be distinguished in mixtures using our devices. This is demonstrated in Fig. 3d, which shows the extinction spectra of two gas mixtures, one containing SO_2_ and N_2_O, another one consisting of SO_2_, N_2_O, and NO_2_. The original extinction spectra are presented in Supplementary Figure 14. These results confirmed that the rotational–vibrational fingerprint peaks of each molecular species in the gas mixtures could be clearly identified using our graphene nanoribbons devices.

### Monitoring of gaseous components during chemical reactions

**Fig. 4:**
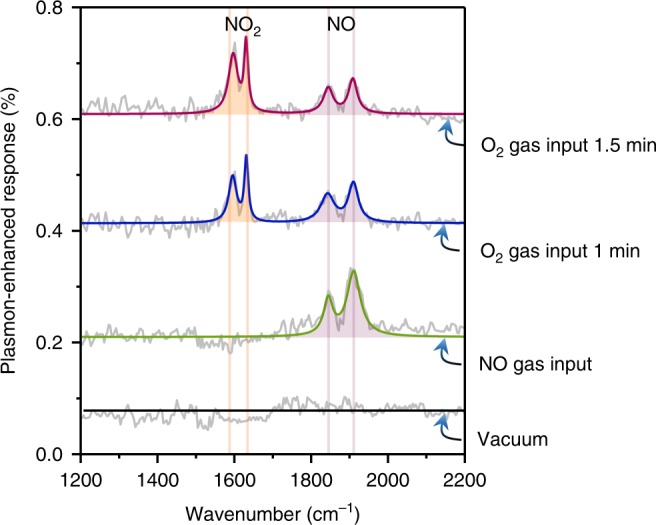
Identification of gas molecules during chemical reactions. From bottom to top: plasmon-enhanced molecular signals in a chamber filled with no gas (i.e. vacuum), 1 min and 1.5 min after O_2_ input. We corroborated the direct observation of the oxidation of NO to NO_2_

Real-time and accurate identification of gas molecules is extremely useful in a range of applications, such as monitoring of gas-phase chemical reactions. We successfully demonstrate this concept for an NO oxidation reaction. The measured plasmon-enhanced response in this reaction is shown in Fig. 4 (extracted from the extinction spectra in Supplementary Figure 15). First, the chamber was filled with NO gas, which was clearly identifiable based on the rotational–vibrational peak positions at 1838 and 1906 cm^−1^ (see green curve). Then O_2_ was injected into the chamber. As shown in the extinction spectrum recorded after 1 min, the signal intensity of NO decreased to ~60%, while a new pair of peaks appeared at 1590 and 1610 cm^−1^ (blue curve in Fig. 4). These new peaks, which coincide with the rotational–vibrational modes of NO_2_ (Fig. 3b), clearly confirm the production of NO_2_ due to chemical reactions between NO and O_2_. As input of O_2_ gas continued, the NO_2_ response increased and the signal intensity of NO decreased, as shown in the spectrum recorded 30 s later (burgundy curve in Fig. 4). These real-time measurements are highly selective, enabling direct observation of chemical reactions that holds great potential for use in applications requiring analysis of in situ chemical reactions.

## Discussion

In the present study, we successfully demonstrated label-free identification of the gases NO_2_, N_2_O, NO, and SO_2_ using graphene plasmons. The large physisorption of gas molecules on graphene nanoribbons combined with the ultra-confined plasmonic near-fields were critical for overcoming the extremely weak dielectric response of gas molecules and achieving high sensitivity of 800 zeptomole molecule per μm^2^.

The sensitivity of our plasmonic devices was closely correlated with gas adsorption on graphene. Various adsorption mechanisms such as physisorption^15,42,47^, optical trapping^48–50^, and static dielectrophoretic forces^51,52^ have been previously demonstrated to support increasing gas concentrations on graphene structures. Moreover, the ultra-strong optical-field confinement of graphene plasmons could potentially lead to large gradient optical forces^48,53^ and serve as resonant optical tweezers that drag gas molecules onto the graphene surface. However, given the power density of the infrared beam, the optical force calculated in this experiment was too weak to modify the gas distribution (see details in Supplementary Figure 16 and Note 9). Moreover, for the CaF2 thickness used here and the applied bias range, the electrostatic force is also expected to be weak to initiate trapping. Physisorption was thus possibly the primary mechanism responsible for gas molecule accumulation on the graphene layer through image attraction force and defect adsorption as discussed above. Clear evidence in support of physical adsorption rather than chemical adsorption was provided by the fact that the detected rotational–vibrational modes were identical to those in pristine gases. This is typically not observed for chemical adsorption because chemical bonding to graphene modifies these rotational–vibrational modes. Additional evidence was provided by relatively fast molecular desorption observed with N_2_ flow through the chamber (see Fig. 2).

It is worth noting that further improvements of the sensor can effectively enhance its sensitivity potentially for broader applications. For example, higher crystal quality and higher mobility graphene can effectively increase the quality factor and field enhancement effect of graphene plasmons as shown in Supplementary Figure 17, thus resulting in a dramatic increase in extinction intensity, which further facilitates larger enhancement of the signal for molecular detection. Moreover, multi-layer graphene can generate higher field strengths and additionally produce adsorption more molecules. Indeed, we present further experiments in Supplementary Figure 18 in which bilayer graphene is shown to increase the detection limit to 800 ppm of SO_2_ molecules. A suitably designed resonant microcavity for perfect light absorption in graphene should also enable further increase in the detection limit^54,55^. Further enhancement in graphene confinement can also be achieved by placing graphene in proximity to a metallic surface, hence inducing acoustic plasmons with enhanced sensitivity of vibrational modes of adsorbed layers^56^.

In conclusion, we performed real-time and label-free gas identification by using graphene plasmons, which can unambigously distinguish between different types of gases even when the gas molecules have similar compositions. This advanced feature opens exciting prospects for gas sensing and identification in diverse applications, including the detection of dilute contaminants and monitoring of trace chemical reactions. The sensitivity and time resolution of our devices could be further improved in the future by designing sensors that exploit optical gradients^50^ and dielectrophoretic forces^51,52^, as well as large variations in physisorption with changes in temperature.

## Methods

### Nanofabrication of graphene plasmon devices

Chemical vapor deposition graphene was first transferred onto a 300-nm SiO_2_/500-μm SiO_2_ substrate using a common wet method^57^. A 120-nm poly(methyl methacrylate) (950 K) film was spin-coated onto the sample. The nanoribbon arrays were patterned in graphene using electron-beam lithography (Vistec 5000+ES, Germany) and oxygen plasma-etching at 5 Pa and 80 W for 10 s (SENTECH, Germany). Two Cr (5 nm)/Au (60 nm) electrode patterns were fabricated using a second electron-beam lithography cycle combined with electron beam evaporation (OHMIKER-50B, Taiwan). The growth rate of CaF_2_ film was 0.5 Å/s at 100 °C in the a high vacuum of ~10^–6^ Torr. The graphene device on SiO_2_ was transferred onto the CaF_2_/Si substrate and then annealed at 200 °C for 5 h.

### Characterization of graphene plasmon devices

The morphologies and thicknesses of the fabricated graphene nanoribbons were characterized by scanning electron microscopy (Hitachi S-4800) and atomic force microscopy (Neaspec s-SNOM). The quality of the graphene and defect density of the nanoribbons were measured by Raman spectroscopy (Horiba Jobin Yvon LabRAM HR800) with a laser excitation at 514 nm. Electrical properties were determined using a semiconductor parameter analyser (Agilent 4294 A).

### FTIR microscopy measurements

A home-made gas chamber was designed to meet the requirements for electrical measurements and transmission spectrum detection simultaneously. A high-precision piezometer and mass-flow controller with a 50–400 sccm flow at different times (in minutes) were used to control the partial pressure of the analyte gases. For the transmission measurements (Thermo Fisher Nicolet iN10), the excitation light was broadband. For all the extinction spectra in this work, we first recorded the transmission spectrum *T*_CNP_ of the graphene array at the CNP, and then the extinction spectra *η* for in situ nanoribbon doping at *E*_F_ were calculated as 1-*T*_Vg_/*T*_CNP_.

### Electromagnetic simulations and theory

Electromagnetic simulations were conducted using the commercial field solver, COMSOL Multiphysics, RF module. The graphene optical response is described via the Drude model. The gas parameters including oscillator strength and broadening corresponding to each FTIR peak for the gas molecules were extracted from fitting to the measured data.

## Supplementary information

Supplementary Information

## Data Availability

The data that support the findings of this study are available from the corresponding author upon request.
