# Supplementary material for: Gas identification with graphene plasmons
Source: Nat Commun. 2019 Mar 8;10:1131. doi: 10.1038/s41467-019-09008-0 (PMC6408516; doi:10.1038/s41467-019-09008-0)
Supplement: Supplementary file 1 — Supplementary Information [file 41467_2019_9008_MOESM1_ESM.docx]

**Gas identification with graphene plasmons**

Hai Hu et. al.

**Supplementary Information**

**Supplementary Table 1.**

Comparison of graphene plasmons for gas detection with electronic devices^1-7^. ΔR is the response signal, which is the ratio of resistance before and after sensing.

**Supplementary Table 2.**

Comparison of graphene plasmons for gas detection with metal plasmon sensors^8-15^. Δλ is the response signal, which is the difference of plasmon wavelength before and after sensing.

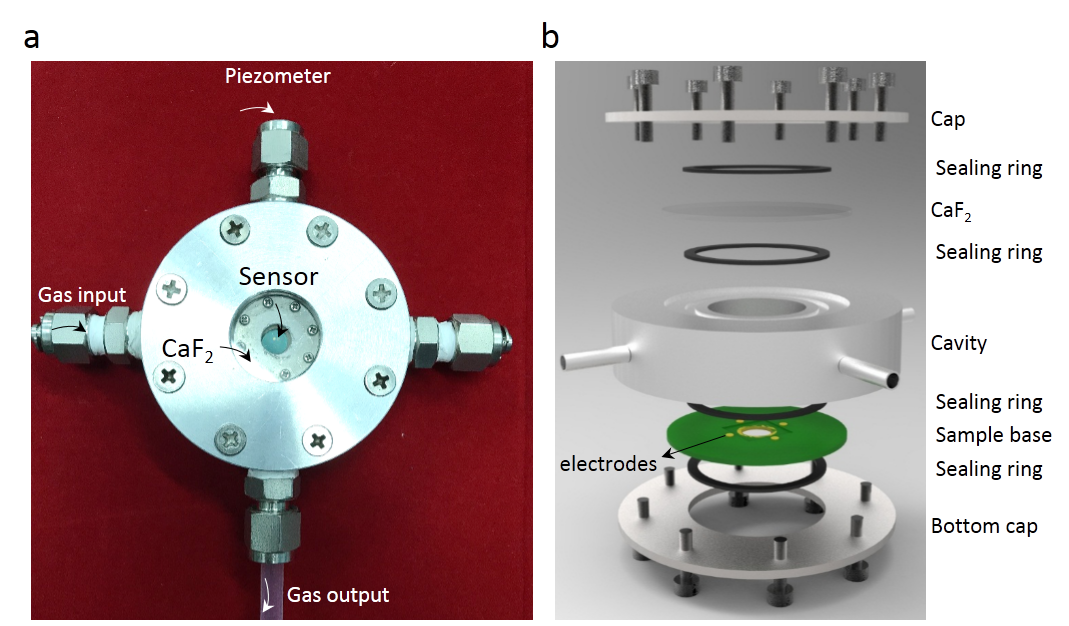


**Supplementary Figure 1.** **(a)** A photograph of the home-made chamber for gas identification with graphene plasmons. **(b)** Schematic of components in the chamber.


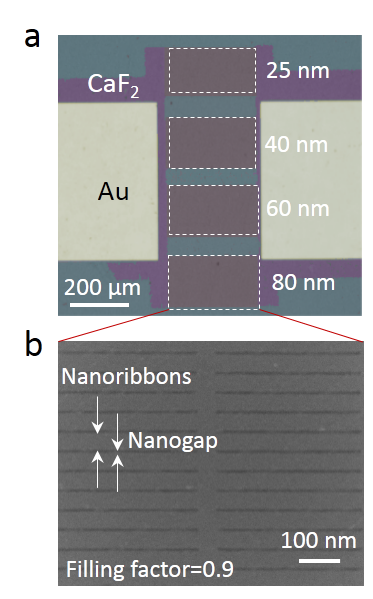


**Supplementary Figure 2. (a)** SEM image of the graphene device. Nanoribbon arrays are indicated by dashed rectangles. **(b)** Enlarged SEM image of a typical graphene nanoribbon array.


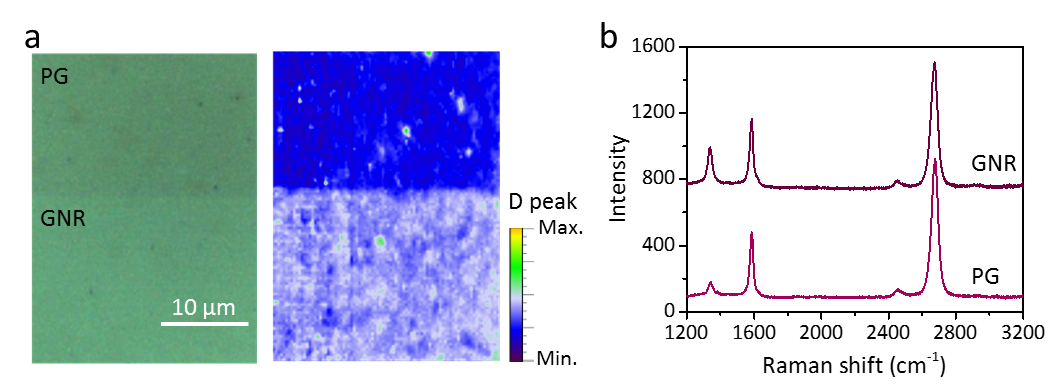


**Supplementary Figure 3.** Optical image (left) and Raman D peak mapping (right) of an unpatterned graphene sheet and graphene nanoribbons (GNR).

**Supplementary Note 1: Defect density in graphene nanoribbon arrays.** Supplementary Figure 3 shows the Raman D peak mappings of an unpatterned graphene sheet and graphene nanoribbons. The intensity of the Raman D peak of graphene nanoribbons is much stronger than that of the unpatterned graphene sheet, implying that graphene nanoribbons have a higher defect density than unpatterned graphene. The defect density calculated from the expression^16-17^ $n_{D}^{2}\left( cm^{-2} \right)= \frac{5.9\times{10}^{14}}{E_{L}^{4}(eV^{4})}\left[ \frac{I\left( D \right)}{I\left( G \right)} \right]^{-1}$, yields $n_{D} \sim$10^5^ $\mu m$^-2^.

**Supplementary Figure 4.** (a) Illustration of the method of *in situ* measurement of extinction spectra. (b) Original data of a spectrum at *E*_F_=V_CNP_ and another spectrum at *E*_F_=V_g_. (c) Extinction spectrum calculated as 1-T_CNP_/T_Vg_.

**Supplementary Note 2:** Gas detection and identification measurements with our graphene nanostructures were performed by recording their IR transmission spectra using Fourier transform infrared spectroscopy (FTIR). First, the extinction spectra (*T*_CNP_) of the graphene nanoribbon array at the charge neutral point (CNP) (i.e., *E*_F_=0) were collected. Then we only changed *E*_F_ of the graphene nanoribbon array, and *in situ* measured the extinction spectra T*_Vg_*. The electromagnetic response of the graphene plasmon at each value of *E*_F_ was obtained from the extinction spectrum (i.e., calculating the extinction as $\eta$ =1-T*_Vg_ /*T_CNP_). Since the measurement process is *in situ* and only the Fermi energy of graphene changes in the two measurements, the background IR extinction including gas molecules in the gas region and the substrates were all cancelled out.


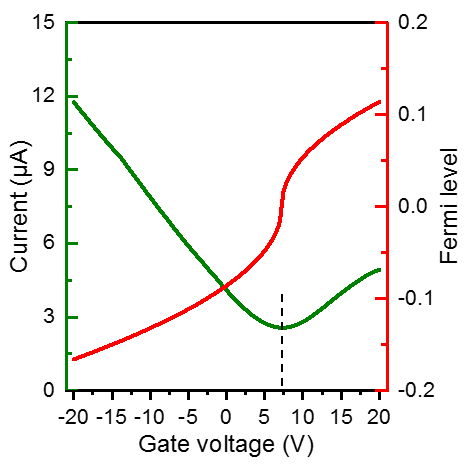


**Supplementary Figure 5.** Transfer curve (green) of our graphene/CaF_2_ device. The gate voltage that corresponds to the charge neutrality point (CNP, V_CNP_, indicated by a dashed line) is 7.2 V. The red curve shows the dependence of the graphene Fermi energy on gate voltage.

**Supplementary Note 3: Calculation of Fermi energy.** The dependence of the carrier density on the gate voltage satisfies the equation

, (S1)

where $V_{g}$ is the gate voltage, $V_{CNP}$ is the charge-neutrality-point voltage, υ_F_=1×10^6^ m/s is the Fermi velocity, n is the carrier density, and C_g_ is the capacitance of the gate dielectric. For a CaF_2_ thin film, using a relative dielectric constant of 7.3 and a thickness of 400 nm, the capacitance is C_g_ =0.015 μFcm^-2^. Combined with the relation between *E*_F_ and the carrier density of graphene $E_{F}=\hbar\nu_{F}\sqrt{\pi n}$, we obtain a relation between *E*_F_ and the gate voltage as shown in Supplementary Figure 5, where *E*_F_ is plotted as a function of gate voltage V_g_.

The FET mobilities of hole and electron carriers in our graphene devices can be calculated as

$$\mu=\frac{dG/dVg}{Cg}\times\frac{L}{W}$$

where *C*_g_ is the unit-area capacitance of the back-gate, *L* and *W* are the channel length and width of the suspended graphene between source and drain, *G* is the conductance of the device, and *dG*=*dVg* is the transconductance for hole or electron branches. The mobilities of holes and electrons for the device are estimated to be 312 cm^2^/(Vs).

**
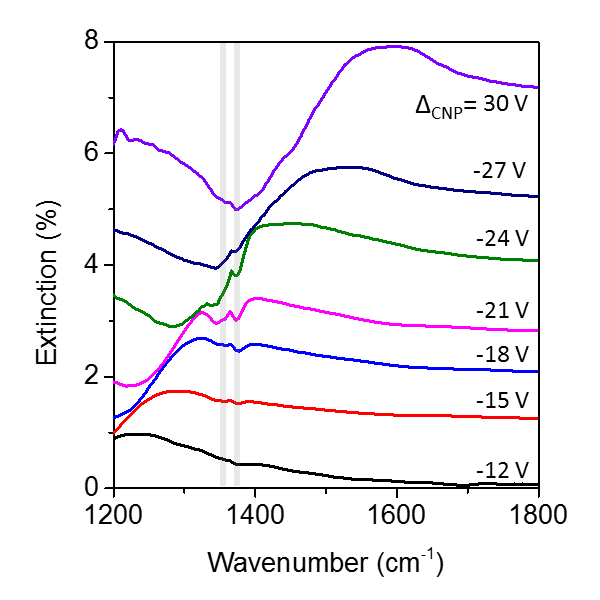
**

**Supplementary Figure 6.** Electrically tunable graphene plasmons for gas detection. The graphene plasmon resonance frequency can be continuously tuned from 1243 to 1588 cm^-1^ by changing *Δ*_CNP_ from -12 to -30 V. The signal strengths of gas molecular vibrational modes gradually increase when *Δ*_CNP_ varies from -12 to -21 V, which is expected because the graphene plasmon resonance frequency is tuned close to these modes. The signal strengths of gas molecular vibrational modes gradually decrease when *Δ*_CNP_ decreases from -21 to -30 V, which is also expected because the graphene plasmon resonance frequency is tuned away from these modes. The strong dependence of the strength of the dips in the spectra on voltage in this experiment also reveals the coupling between graphene plasmons and gas molecules.


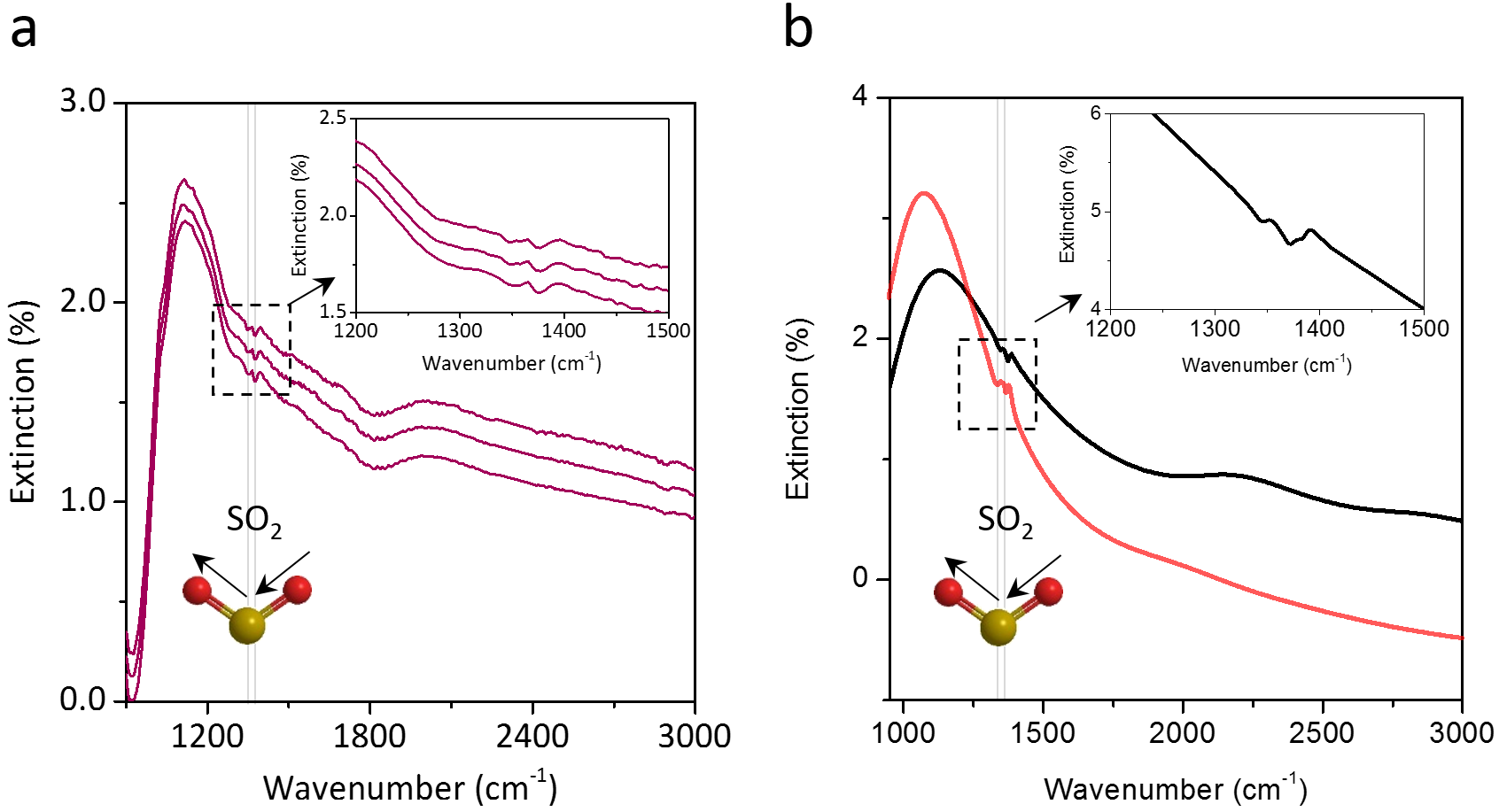


**Supplementary Figure 7.** **(a)** Extinction spectra of graphene plasmons coupled to SO_2_ gas (4000 ppm in N_2_). The width of the graphene nanoribbons is designed to be *W*= 100 nm and the voltage is$\Delta$*V*_CNP_ = 30 V. The extinction spectra are measured three times. **(b)** Simulated extinction spectra in the presence of SO_2_ gas with both COMSOL (black) and analytical model (red). This is obtained assuming: *W_eff_*=*W*-30 nm=70 nm. *E*_F_=0.3 eV, and a graphene mobility of 300 cm^2^/(V s).

**Supplementary Figure 8. (a)** Direct measurement of FTIR absorption spectra of SO_2_ with different concentrations without GNRs. **(b)** Extracted oscillator strength (symbols) and linear fitting (lines) vs concentration.

**Supplementary Note 4: Modeling gas extinction spectra.** We use COMSOL Multiphysics [*COMSOL Multiphysics® v. 5.2. www.comsol.com. COMSOL AB, Stockholm, Sweden.*], RF module in conjunction with a transfer matrix method to obtain the electromagnetic response of the graphene-gas system.^18^  The scattering parameters are extracted from COMSOL for a geometry of (from bottom to top), Si (with relative permittivity 12.11), CaF_2_ (with 400 nm thickness and relative permittivity 1.96), graphene ribbons, adsorbed gas layer (with thickness $d_{l}$), and a $\Delta$ = 2 $\mu m$ thick air layer. The transfer matrix method is then used to compute the extinction of the full geometry including air, the adsorbed gas layer, and the rest of graphene nanoribbons and CaF_2_ film with its transfer matrix elements obtained via converting the scattering parameters extracted from COMSOL.

The graphene conductivity is modeled using the Drude formula $\sigma=iD/(\omega+i\tau^{-1})$, where$D=e^{2}E_{F}/\pi\hbar^{2}$ is the Drude weight, while $\tau$ and $E_{F}$ denote the relaxation time and Fermi energy, respectively.^19^ We use the valuses $E_{F}=$ 0.3 eV, $E_{F}^{cnp}$= 0.15 eV and $\tau=$ 10 fs.

The oscillator strength corresponding to each FTIR peak varies with the gas concentration (see Supplementary Figure 8). This variation can be modeled by linear fitting to the measured data: $\Delta\epsilon_{x,i}=p_{x,i}C$, with $x\to P$ or $R$. For the peaks in the FTIR of the SO_2_, the obtained proportionality constants are $4.298\times{10}^{-10}$. These are then used to estimate $\Delta\epsilon_{x,i}$ at $C_{u}$ and $C_{l}$. The linewidth broadening for the P-R peaks are set to $\gamma_{P,1}/\Omega_{P,1} = \gamma_{R,1}/\Omega_{R,1}= 0.025$and $\gamma_{P,2}/\Omega_{P,2}= \gamma_{R,2}/\Omega_{R,2}= 0.01$.

**Supplementary Note 5: Analytical model.** An accurate analytical model for the extinction produced by the graphene ribbons with a thin dense molecular layer is provided by a direct extension of previously reported methods^20, 21^, in which the graphene is described by a local-RPA conductivity $\sigma_{gra}$
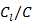
, while the molecules distributed with volume density $C_{l}$ in a layer of thickness $d_{l}$ are effectively assimilated to a surface conductivity $\sigma_{mol}=-i\omega{C_{l}d_{l}\alpha}_{g}$, so that the total conductivity of the graphene with the molecular layer becomes ${\sigma=\sigma}_{gra}+\sigma_{mol}$. The transmission *T* through this layer normalized to the transmission $T_{0}$ in the absence of graphene is then given by $T/{T_{0}}=\left| 1+{iS}/\left( 1/\tilde{\alpha}-G \right) \right|^{2}$ with

$$\tilde{\alpha}=\sum_{j} \frac{-\epsilon{W^{2}A}_{j}}{\frac{i\epsilon W\omega}{\sigma}+\frac{1}{\eta_{j}}}$$

where *j* labels dipole active graphene modes (we include *j*=1-3 in practice);$A_{1}$=0.889, $A_{2}$=0.057, and $A_{3}$=0.019 are transition-strength factors ^21^; $\eta_{1}$=-0.0687, $\eta_{2}$=-0.0184, and $\eta_{3}$=-0.0107 are mode eigenfrequencies; $\epsilon$ is the average permittivity of the substrate and the surrounding gas; and $G=2\pi^{2}/(3{\epsilon a}^{2})+iS$ accounts for inter-ribbon interaction, where *a* is the ribbon period, $S=4\pi^{2}/(na\lambda_{0})$ corrects for radiative losses, and $n$ is the average refractive index of the substrate and surrounding gas.

**Supplementary Figure 9.** Extinction spectra obtained with the plasmon sensor in response to SO_2_ exposure in real time.

**Supplementary Figure 10. (a)** Measured extinction spectra of graphene plasmons with (color curve) and without (black curve) SO_2_ gas molecules. **(b)** Plasmon-enhanced rotational-vibrational response of the SO_2_ molecules. The spectrum is extracted from the extinction spectrum with plasmonic enhancement (colored curve) by subtracting the original (black curve) graphene plasmon spectrum in **(a)**.

**Supplementary Note 6: Extraction of the molecular response from the plasmonic resonance.** In Supplementary Figure 10a, the black curves represent the fitted strength of graphene plasmons without gases using the lineshapes derived from the original plasmonic resonance peaks. We then obtain the signatures of gases on the graphene plasmon by subtracting the original graphene plasmon spectrum (black curves) from their extinction spectra (color curves), following a method described elsewhere.^22, 23^ In Supplementary Figure 10b, we show the plasmon-enhanced rotational-vibrational mode response as extracted from the extinction spectra of Supplementary Figure 10a. Each curve is the average of three measurements, and the grey shaded regions represent error bounds.

**Supplementary Note 7: Caculation of adsorbed concentration**. According to the ideal gas law, we have *PV*=*nRT* and *C_tot_* =*n*/*V* = *P/RT*, where *R* is 82 cm^3^ atm K^-1^ mol^-1^, *P* is 4 atm, *T* is 300 K, so we obtain *C_tot_*=0.00016 mol/cm^3^. Since there is only 0.1% SO_2_ in the carrier N_2_ (1000 ppm), the SO_2_ concentration is *C*=1.6 × 10^-7^ mol/cm^3^. The gas adsorption concentration is C_l_ × d_l_ = 5000 × C × 1 nm =8 × 10^-11^ mol/cm^2^=8 × 10^2^ zmole/µm^2^ (1 zmole =10^-21^ mole).


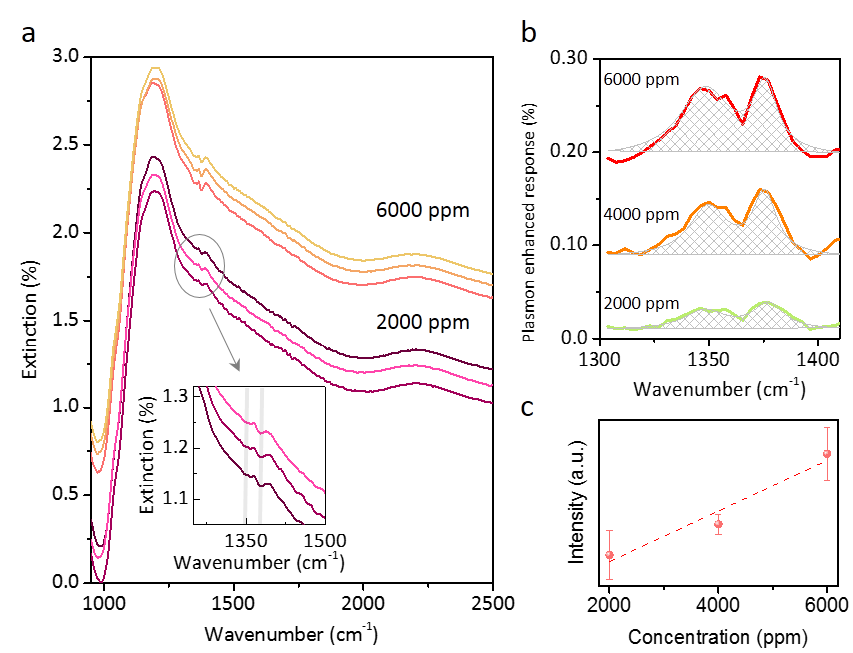


**Supplementary Figure 11.** Spectral response towards different SO_2_ concentrations. **(a)** Extinction spectra of the graphene plasmon coupled with SO_2_ at two typical gas concentrations. **(b)** Extracted plasmon enhanced response of SO_2_ molecules at different gas concentrations. **(c)** Plasmon enhanced SO_2_ signal intensity (i.e., the integrated area of the peaks in (**b**)) as a function of gas concentration.

**Supplementary Note 8: Plasmonic response for different SO_2_ concentrations.** To probe the quantitative performance of the sensor, Supplementary Figure 11 shows the sensing response towards different SO_2_ concentrations. Before conducting each measurement, the gas flow was passed through the chamber for 30 min to reach a maximum adsorption of gas molecules. The sensor is highly sensitive even for 2000 ppm SO_2_ gas. As the SO_2_ concentration increases, the dips are clearly deeper.

**Supplementary Figure 12.** Change of resistance of the graphene plasmonic device in the presence of N_2_O, NO_2_, and NO, respectively.


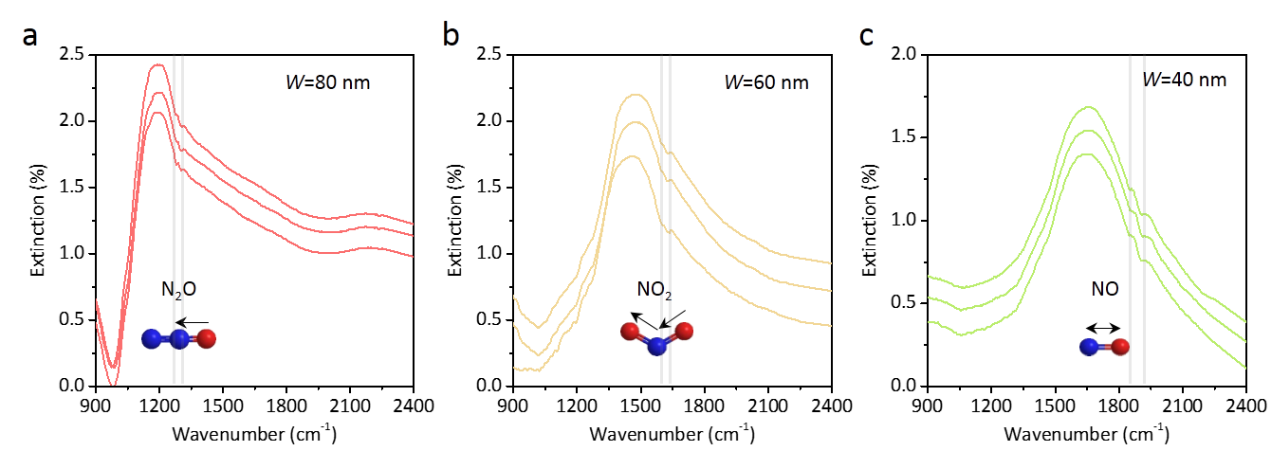


**Supplementary Figure 13.** Identification of different nitrogen oxides. **(a-c)** Extinction spectra of graphene plasmons in the presence of N_2_O, NO_2_, and NO gases, respectively. The respective rotational-vibrational modes are marked by vertical lines. The measurements are repeated three times to guarantee the accuracy of the results.


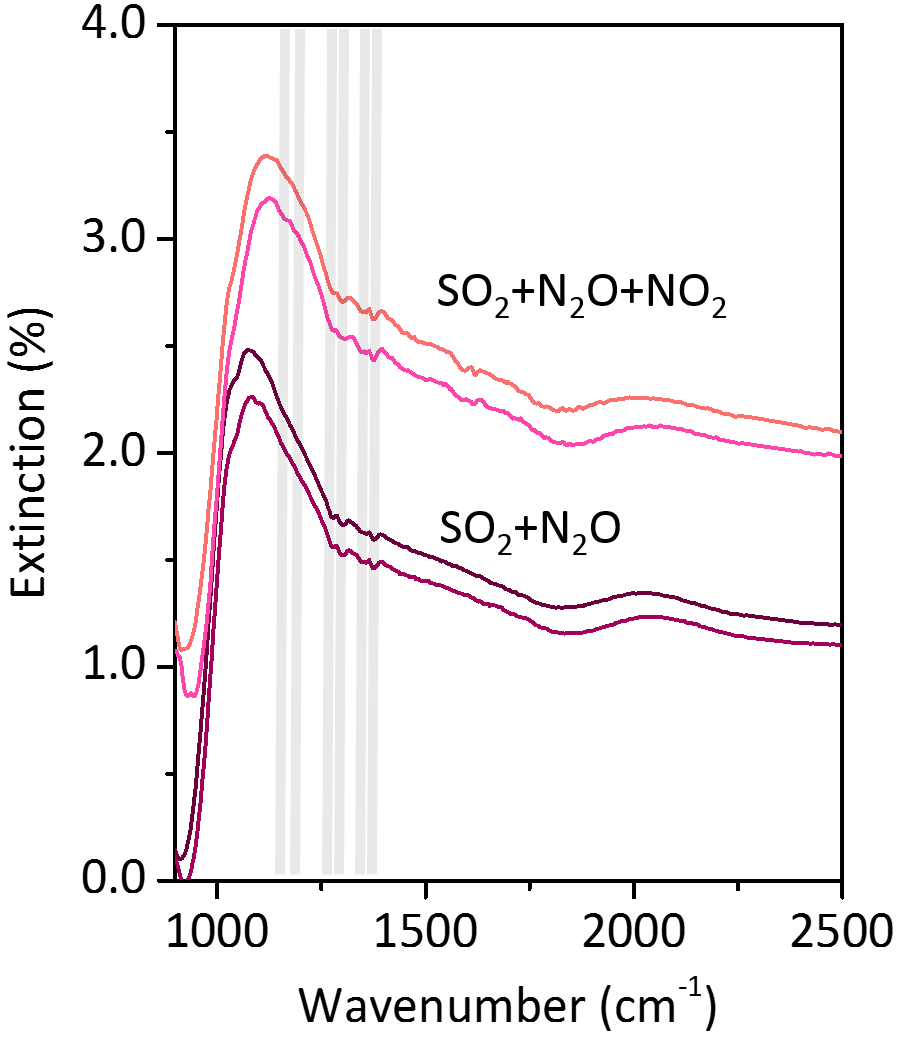


**Supplementary Figure 14.** Detection of different mixtures. Extinction spectra of graphene plasmons in the presence of a gas mixture containing 2500 ppm SO_2_ and 6000 ppm N_2_O, and another mixture containing 4000 ppm SO_2_, 8000 ppm N_2_O, and 5000 ppm NO_2_.


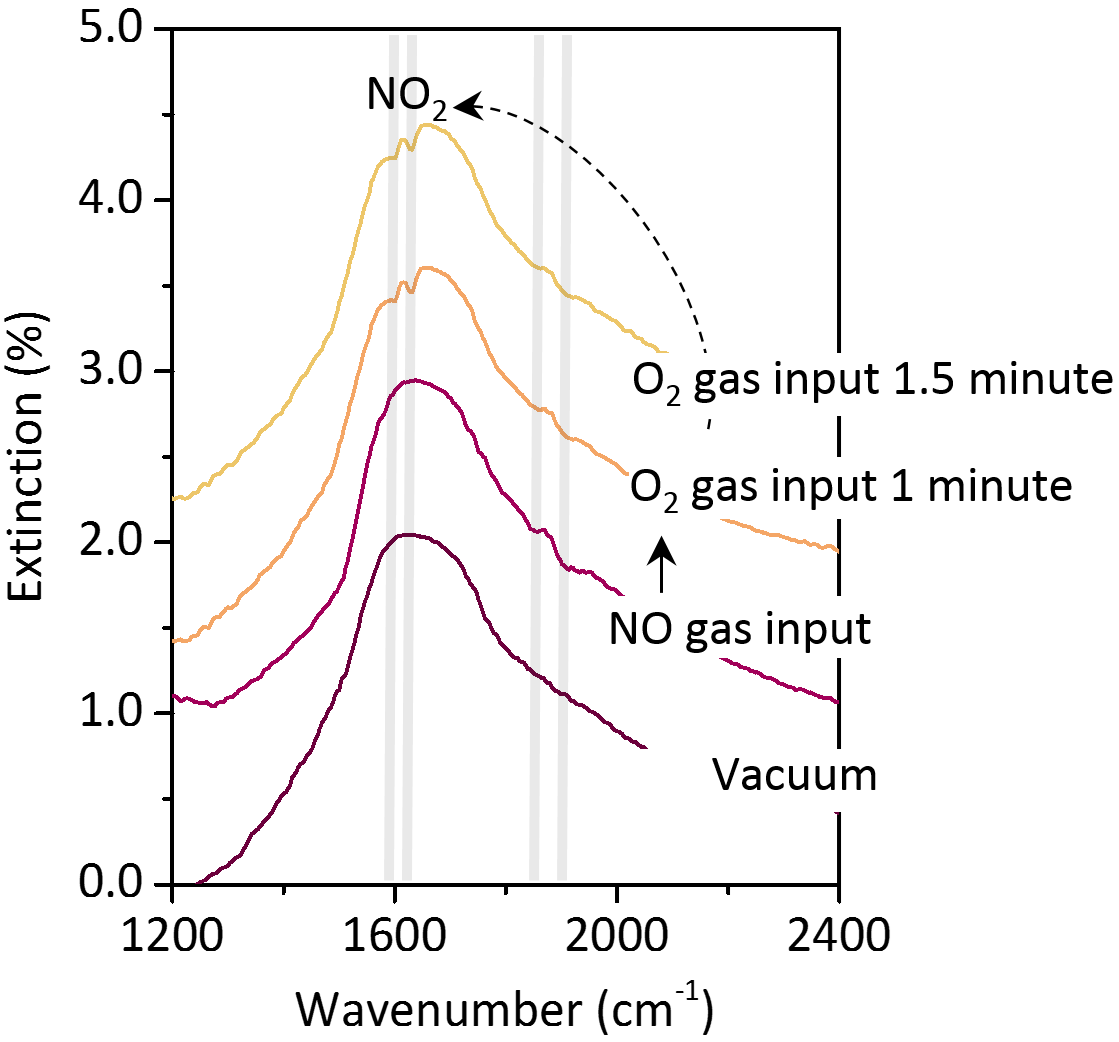


**Supplementary Figure 15.** Direct observation of gas-phase chemical reaction for oxidazion of NO (5000 ppm) by O_2_ to form NO_2_. We show the measured original extinction spectra of graphene plasmons at different temporal stages along the reaction.


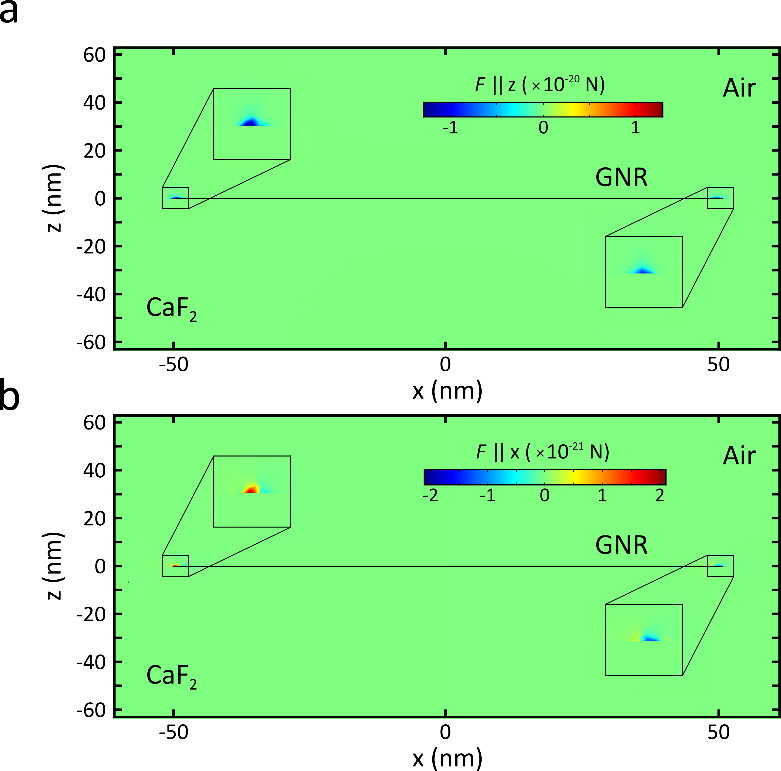


**Supplementary Figure 16.** Spatial distribution of **(a)** *z*- and **(b)** *x*-components of the optical force.

**Supplementary Note 9: Modeling plasmonic optical forces.** The optical force is calculated using the well-known relation $\vec{F}=\frac{1}{4}\mathfrak{R}\left\{ \alpha\right\}\vec{\nabla}$^24^, where $\alpha$ denotes the gas-molecules polarizability, obtained from the dielectric function through the Clausius-Mossotti relation $\alpha=3\epsilon_{0}V \left( \epsilon_{g}\left( C_{t} \right)-\epsilon_{0} \right)/\left( \epsilon_{g}\left( C_{t} \right)+2\epsilon_{0} \right)$. Force calculations are performed using COMSOL Multiphysics, RF Module. As shown in Supplementary Figure 16, plasmon-induced optical forces are significant only in the 1 nm range of ribbon edges, and tend to trap molecules in close vicinity of the edges. For SO_2_, the polarizability is of the order of 10^-38^ F.m^2^. With a power density of 0.05 kW/cm^2^ used in the measurements, the maximum optical force which occurs at plasmon resonance is of the order of 10^-20^ N. For stable trapping of SO_2_ molecules (i.e. to effectively suppress their Brownian motion) the potential energy defined as $U= -\int\vec{F}.d\vec{r}$ must surpass the thermal energy.^25^ The latter translates into the following criterium: for optical forces to play a role in SO_2_ redistribution, forces of at least 10^-13^ N are needed. This condition is not fulfilled in our experiments.


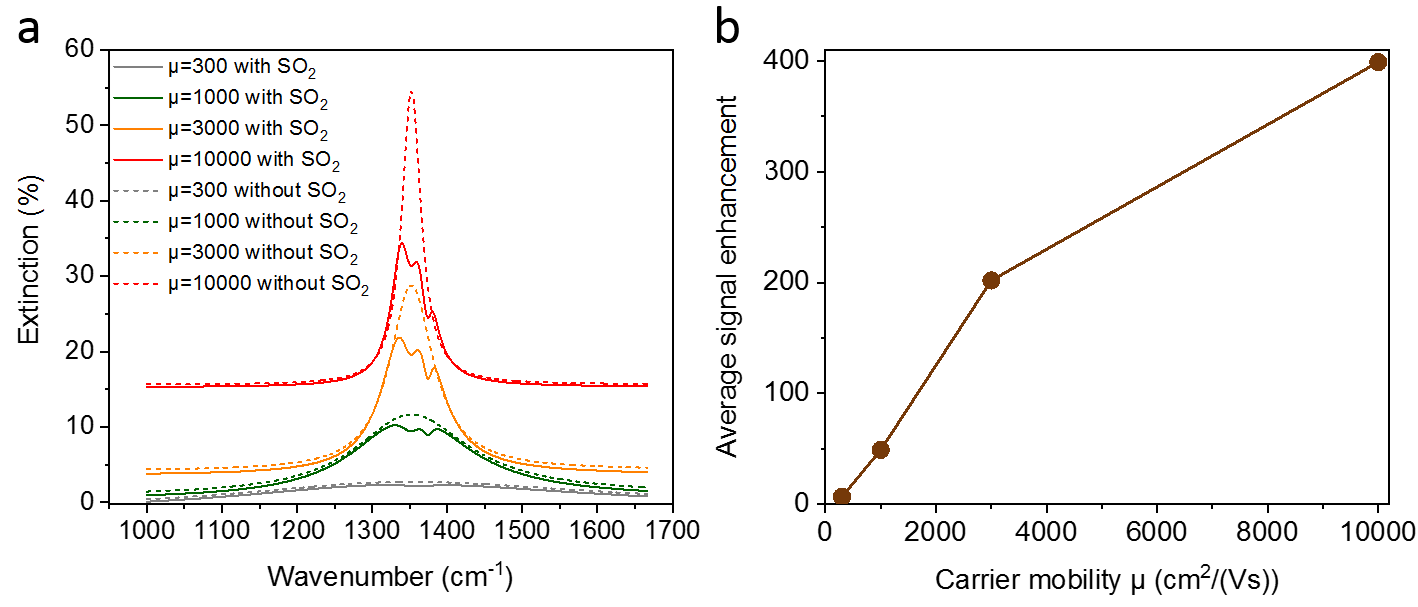


**Supplementary Figure 17**. (a) Simulation of extinction spectra of graphene plasmons before (dashed curves) and after (solid curves) coating with 8 nm thick SO_2_ gas molecules at various values of the carrier mobility. (b) Signal enhancements (variation of extinction) at various values of carrier mobility, extracted from (a).


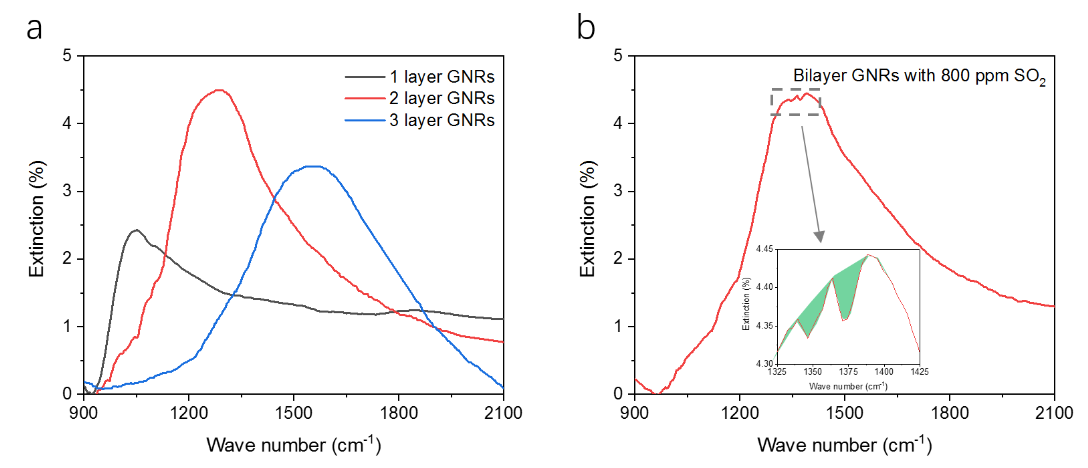


**Supplementary Figure 18.** (a) Extinction spectra of one-, two-, and three-layer graphene nanoribbon arrays showing how the plasmon resonance frequency increases with the number of layers. (b) Highly sensitive detection experiments of 800 ppm SO_2_ molecular vibrations with bilayer graphene nanoribbons.

**Supplementary References**

1. Schedin F*, et al.* Detection of individual gas molecules adsorbed on graphene. *Nat Mater* **6**, 652-655 (2007).

2. Kulkarni GS, Reddy K, Zhong Z, Fan X. Graphene nanoelectronic heterodyne sensor for rapid and sensitive vapour detection. *Nat Commun* **5**, 4376 (2014).

3. Dua V*, et al.* All-organic vapor sensor using inkjet-printed reduced graphene oxide. *Angew Chem Int Ed Engl* **49**, 2154-2157 (2010).

4. Paul RK, Badhulika S, Saucedo NM, Mulchandani A. Graphene nanomesh as highly sensitive chemiresistor gas sensor. *Anal Chem* **84**, 8171-8178 (2012).

5. Cho SY*, et al.* Superior Chemical Sensing Performance of Black Phosphorus: Comparison with MoS2 and Graphene. *Adv Mater* **28**, 7020-7028 (2016).

6. Yasaei P*, et al.* Chemical sensing with switchable transport channels in graphene grain boundaries. *Nat Commun* **5**, 4911 (2014).

7. Cui S*, et al.* Ultrahigh sensitivity and layer-dependent sensing performance of phosphorene-based gas sensors. *Nat Commun* **6**, 8632 (2015).

8. Kreno LE, Hupp JT, Van Duyne RP. Metal− organic framework thin film for enhanced localized surface plasmon resonance gas sensing. *Anal Chem* **82**, 8042-8046 (2010).

9. Cittadini M, Bersani M, Perrozzi F, Ottaviano L, Wlodarski W, Martucci A. Graphene oxide coupled with gold nanoparticles for localized surface plasmon resonance based gas sensor. *Carbon* **69**, 452-459 (2014).

10. Powell AW, Coles DM, Taylor RA, Watt AAR, Assender HE, Smith JM. Plasmonic Gas Sensing Using Nanocube Patch Antennas. *Adv Opt Mater* **4**, 634-642 (2016).

11. Allsop T*, et al.* Photonic gas sensors exploiting directly the optical properties of hybrid carbon nanotube localized surface plasmon structures. *Light Sci Appl* **5**, e16036 (2016).

12. Bingham JM, Anker JN, Kreno LE, Van Duyne RP. Gas sensing with high-resolution localized surface plasmon resonance spectroscopy. *J Am Chem Soc* **132**, 17358-17359 (2010).

13. Tang ML, Liu N, Dionne JA, Alivisatos AP. Observations of shape-dependent hydrogen uptake trajectories from single nanocrystals. *J Am Chem Soc* **133**, 13220-13223 (2011).

14. Liu N, Tang ML, Hentschel M, Giessen H, Alivisatos AP. Nanoantenna-enhanced gas sensing in a single tailored nanofocus. *Nat Mater* **10**, 631-636 (2011).

15. Farmer, D. B., Avouris, P., Li, Y., Heinz, T. F., Han, S.-J. Ultrasensitive Plasmonic Detection of Molecules with Graphene. Acs Photon. 3, 553-557 (2016).

16. Ferrari AC, Basko DM. Raman spectroscopy as a versatile tool for studying the properties of graphene. *Nat Nanotech* **8**, 235-246 (2013).

17. Lucchese MM*, et al.* Quantifying ion-induced defects and Raman relaxation length in graphene. *Carbon* **48**, 1592-1597 (2010).

18. Markos P, Soukoulis CM. Wave propagation: from electrons to photonic crystals and left-handed materials. *Princeton University Press* (2008).

19. Zhan TR, Shi X, Dai YY, Liu XH, Zi J. Transfer matrix method for optics in graphene layers. *J Phys-condens Mat* **25**, (2013).

20. de Abajo FJG. Graphene Plasmonics: Challenges and Opportunities. *Acs Photon* **1**, 135-152 (2014).

21. Yu R, Cox JD, Saavedra JRM, García de Abajo FJ. Analytical Modeling of Graphene Plasmons. *Acs Photon* **4**, 3106-3114 (2017).

22. Liu F, Cubukcu E. Tunable omnidirectional strong light-matter interactions mediated by graphene surface plasmons. *Phys Rev B* **88**, 115439 (2013).

23. Shi Z*, et al.* Gate-dependent pseudospin mixing in graphene/boron nitride moire superlattices. *Nat Phys* **10**, 743-747 (2014).

24. Jonáš A, Zemanek P. Light at work: The use of optical forces for particle manipulation, sorting, and analysis. *Electrophoresis* **29**, 4813-4851 (2008).

25. Shoji T, Tsuboi Y. Plasmonic optical tweezers toward molecular manipulation: tailoring plasmonic nanostructure, light source, and resonant trapping. *J Phys Chem Lett* **5**, 2957-2967 (2014).
